# Wavelet Mutation with Aquila Optimization-Based Routing Protocol for Energy-Aware Wireless Communication

**DOI:** 10.3390/s22218508

**Published:** 2022-11-04

**Authors:** Someah Alangari, Marwa Obayya, Abdulbaset Gaddah, Ayman Yafoz, Raed Alsini, Omar Alghushairy, Ahmed Ashour, Abdelwahed Motwakel

**Affiliations:** 1 Department of Computer Science, College of Computing and Information Technology, Shaqra University, Shaqra 11961, Saudi Arabia; 2 Department of Biomedical Engineering, College of Engineering, Princess Nourah bint Abdulrahman University, P.O. Box 84428, Riyadh 11671, Saudi Arabia; 3 Department of Computer Sciences, College of Computing and Information System, Umm Al-Qura University, Mecca 24382, Saudi Arabia; 4 Department of Information Systems, Faculty of Computing and Information Technology, King Abdulaziz University, Jeddah 21589, Saudi Arabia; 5 Department of Information Systems and Technology, College of Computer Science and Engineering, University of Jeddah, Jeddah 21589, Saudi Arabia; 6 Department of Engineering Mathematics and Physics, Faculty of Engineering and Technology, Future University in Egypt, New Cairo 11845, Egypt; 7 Department of Computer and Self Development, Preparatory Year Deanship, Prince Sattam bin Abdulaziz University, Al-Kharj 16278, Saudi Arabia

**Keywords:** wireless communication, routing protocol, wavelet mutation, Aquila optimizer, wireless sensor networks

## Abstract

Wireless sensor networks (WSNs) have been developed recently to support several applications, including environmental monitoring, traffic control, smart battlefield, home automation, etc. WSNs include numerous sensors that can be dispersed around a specific node to achieve the computing process. In WSNs, routing becomes a very significant task that should be managed prudently. The main purpose of a routing algorithm is to send data between sensor nodes (SNs) and base stations (BS) to accomplish communication. A good routing protocol should be adaptive and scalable to the variations in network topologies. Therefore, a scalable protocol has to execute well when the workload increases or the network grows larger. Many complexities in routing involve security, energy consumption, scalability, connectivity, node deployment, and coverage. This article introduces a wavelet mutation with Aquila optimization-based routing (WMAO-EAR) protocol for wireless communication. The presented WMAO-EAR technique aims to accomplish an energy-aware routing process in WSNs. To do this, the WMAO-EAR technique initially derives the WMAO algorithm for the integration of wavelet mutation with the Aquila optimization (AO) algorithm. A fitness function is derived using distinct constraints, such as delay, energy, distance, and security. By setting a mutation probability P, every individual next to the exploitation and exploration phase process has the probability of mutation using the wavelet mutation process. For demonstrating the enhanced performance of the WMAO-EAR technique, a comprehensive simulation analysis is made. The experimental outcomes establish the betterment of the WMAO-EAR method over other recent approaches.

## 1. Introduction

**Figure 1:**
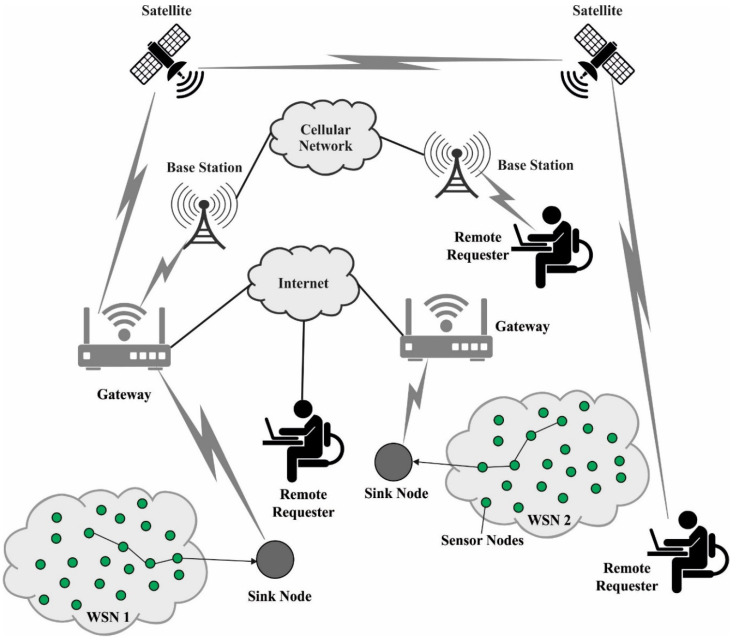
Routing protocol for WSNs.

With the rapid advancement in Future Internet technologies, the mobile Internet, the Internet of Things (IoT), the physical world, and the sensor cloud are regularly getting more connected and moving faster toward the always-connected model [1]. In this model, easily configurable, wirelessly connected, and cheap sensors can sense and gather environmental information anywhere and anytime [2]. Unfortunately, the cheapness and higher availability of sensing techniques could only soften the barrier to truly supporting the level of resilience, scalability, and reliability needed for these models to be global. Efficient software methods and a dearth of accurate simulation models specifically intended for wireless sensor networks (WSNs) result in a lack of reliable algorithms, whose accessibility is crucial for setting up future deployment in better conditions [3]. For instance, systematic consideration of the impacts of network dynamicity, channel interference, and node mobility are key points not sufficiently taken into account yet, preventing or weakening the appropriate operation of the resultant network [4]. Therefore, to smash those barriers, we need fast, accurate, and efficient techniques, particularly to handle communication and judiciously manage the tiny computation, memory, and battery resources of the sensor nodes (SNs) in the network [5]. Figure 1 depicts the overview of a routing protocol for WSNs.

In WSNs, numerous types of research have involved routing strategy, corporeal design, procedure for sensing capability, power management, and security issues of SNs. The lifetime of SNs is the major problem for WSNs, since SNs have constrained energy resources. The routing algorithm has played a crucial role in the SN’s lifetime [6]. Routing in WSNs is different from other wireless networks due to unique properties of SNs, such as processing accomplishment, energy constraints, communication of gathered data from more than one node to a single base station, random deployment of SNs, the improbability of global addresses, and so on [7]. To accommodate this, distinct kinds of routing approaches have been designed. The final objective is to maximize the overall network lifetime and accomplish energy efficiency. The lifetime of SNs is based on the battery life that gives power to SNs, where the lifespan of the entire network is dependent. A power resource supplies energy to the transceiver, memory unit, and sensing unit [8]. The memory unit is applied for the storage of application-related data and information on device identification, the sensing unit has a sensor for capturing information from the environment, and the transceiver takes the responsibility for the reception and transmission of information. Fast energy dissipation in SNs making them lifeless is a crucial issue in the arena of WSNs [9]. It is noted that ineffective routing protocol causes fast dissipation of batteries. Therefore, it is a fundamental prerequisite to use and design energy-effective routing algorithms in WSNs that would rise their [10].

This article introduces a wavelet mutation with Aquila optimization-based routing (WMAO-EAR) protocol for wireless communication. The presented technique aims to accomplish energy-aware routing in WSNs. To do this, it initially derives the WMAO algorithm for the integration of wavelet mutation with the Aquila optimization (AO) algorithm. A fitness function (FF) is derived using distinct constraints, such as delay, energy, distance, and security. Once the mutation probability P has been set, every individual next to the exploitation and exploration process has the probability of mutation using the wavelet mutation process. For establishing the enhanced performance of the WMAO-EAR technique, a comprehensive simulation analysis is made.

## 2. Literature Review

Jagadeesh and Muthulakshmi [11] examined a hybrid metaheuristic (MH) algorithm-based clustering with multihop routing (MHR) (HMA-CMHR) protocol for WSNs. The projected method integrated data transmission, node initialization, clustering, and routing. Initially, the HMA-CMHR algorithm utilized a quantum harmony search algorithm (QHSA)-based clustering procedure for choosing a better subset of CHs. Secondarily, the improved cuckoo search (ICS) technique-based route approach was utilized for a better optimum selection of routes. Gupta and Saha [12] presented a hybrid MH approach where optimum features of ABC and differential evolution (DE) are integrated to measure an optimum group of load-balancing (LB) CHs. Regarding energy efficiency and LB clustering, a new objective function has been developed based on average energy, intracluster distance, and delay parameter. An ABC-based MH technique was presented to the dynamic relocalized mobile sink in a cluster-based network structure.

Al-Otaibi et al. [13] developed a hybridization of the MH cluster-based routing (HMBCR) approach for WSNs. The HMBCR approach primarily contained a brainstorm optimization with levy distribution (BSO-LD)-based clustering employing an FF integrating four parameters: network load, distance to neighbors, energy, and distance to BS. The WWO-HC-based routing method was implemented for optimum route selection. Subramani et al. [14] concentrated on MH-based clustering with routing protocol for UWSNs—MCR-UWSNs. The purpose of the aforementioned system is to choose an effectual group of CHs and route to target. Lakshmanna et al. [15] presented an improved MH-driven energy-aware cluster-based routing (IMD-EACBR) technique for IoT-supported WSNs. The presented IMD-EACBR approach aimed to achieve maximal energy consumption and network lifespan. To attain this, the IMD-EACBR technique used an improved Archimedes optimized algorithm-based clustering (IAOAC) system for CH selective and cluster organization. The TLBO-based MHR (TLBO-MHR) approach has been applied to optimal selective routes to targets.

Srikanth et al. [16] established an MH optimized enabled unequal clustering with MHR protocol (MOUC-MRP) for WSNs. The MOUC-MRP system’s purpose is for selecting CHs and optimum routes to target from WSNs. The harmony search (HS) route-selective manner was developed to an optimum route solution. Mann and Singh [17] examined an improved ABC (iABC) MH with an enhanced searching formula to improve their exploitation abilities and increase the global convergence of the presented MH. An enhanced population-sampling approach was established with Student’s-t distribution that needs only one control parameter for computing and storing, then enhances the efficacy of presented MH.

## 3. The Proposed Model

In this study, a new WMAO-EAR algorithm is proposed for effectual and energy-aware wireless communication. The presented WMAO-EAR technique aims to accomplish energy-aware routing in WSNs.

### 3.1. System Model

Approaches utilized to carry out the presented routing protocol were the network model, energy model, and energy-dissipation model [18]. In the following section, a detailed description of all the models is given.

#### 3.1.1. Energy Model

WSNs involve link heterogeneity, energy heterogeneity, and computational heterogeneity. Energy heterogeneity is regarded as the most important to ensure optimal network performance. In the study, we discussed three levels of energy heterogeneity: advanced, normal, and intermediate nodes. Intermediate node initial energy is between advanced and normal node initial energy, given that m and b represent the percentage of advanced nodes and intermediate nodes correspondingly. Advanced node energy is *α* times more than that of the intermediate and normal node energy is *β* times more than normal node energy. Now, the β is connected with *α* via β=α/2. With $E_{N},$ $E_{I},$ and $E_{A}$ signifying the initial energy of normal, intermediate, and advanced nodes, then energy relationships are formulated, using:

(1) $$E_{N}=E_{0}\;$$

(2) $$E_{I}=E_{0}\left(1+\beta \right)$$

(3) $$E_{A}=E_{0}\left(1+\alpha \right)$$

#### 3.1.2. Energy-Dissipation Model

Reception and data transmission are two fundamental functions in WSNs. Usually, the data-communication method expends lots of energy when compared to data reception. Now, energy cost during k-bit data transmission over distance *d* is formulated as follows.

(4) $$E_{Tx}\left(k,\;d\right)≜\{\begin{array}{cc} k\left(E_{elec}+\varepsilon_{fs}d^{2}\right), & if\;d\leq d_{0}\\ k\left(E_{elec}+\varepsilon_{mp}d^{4}\right), & if\;d>d_{0} \end{array}$$

In Equation (4), $d_{0}$ indicates the reference distance and $d_{0}≜\sqrt{\varepsilon_{fs}/\varepsilon_{mp}}.$ Elec shows the per bit energy cost for running the receiver or transmitter circuits, and $\varepsilon_{fs}$ and $\varepsilon_{mp}$ indicate amplification parameters of the transmitter for free space and multipath fading, correspondingly.

Furthermore, energy costs during k-bit data reception are formulated as follows:

(5) $$E_{Rx}\left(k\right)≜kE_{elec}$$

#### 3.1.3. Network Model

Consider that the n sensor is positioned in the region of M size and the sensor is static. Every sensor is regarded as being aware of the identification and location of other SNs. Also, consider that the advanced node’s location is predetermined, while the normal and intermediate nodes are placed randomly. All the nodes transmit information to neighboring CH for data aggregation. The distance between the BS and nodes is $d_{s}\geq d_{s}^{r},\;$where *d* indicates a predetermined range $\left(d_{s}^{r}=10\;m\right)$.

### 3.2. Design of WMAO Algorithm

Abualigah et al. [19] introduced the fundamental formula of the Aquila optimizer (AO). Usually, the AO algorithm mimics the natural behavior of Aquila for catching the prey. Like other metaheuristic (MH) models, AO was a population-based optimization algorithm that takes place by creating an early population X that has N agents. In Equation (6), the mathematical modeling of the algorithm is given.

(6) $$X_{ij}=r_{1}\times \left(UB_{j}-LB_{j}\right)+LB_{j},\;i=1,2,\ldots Nj=1,2,\ldots ,Dim$$

**Figure 2:**
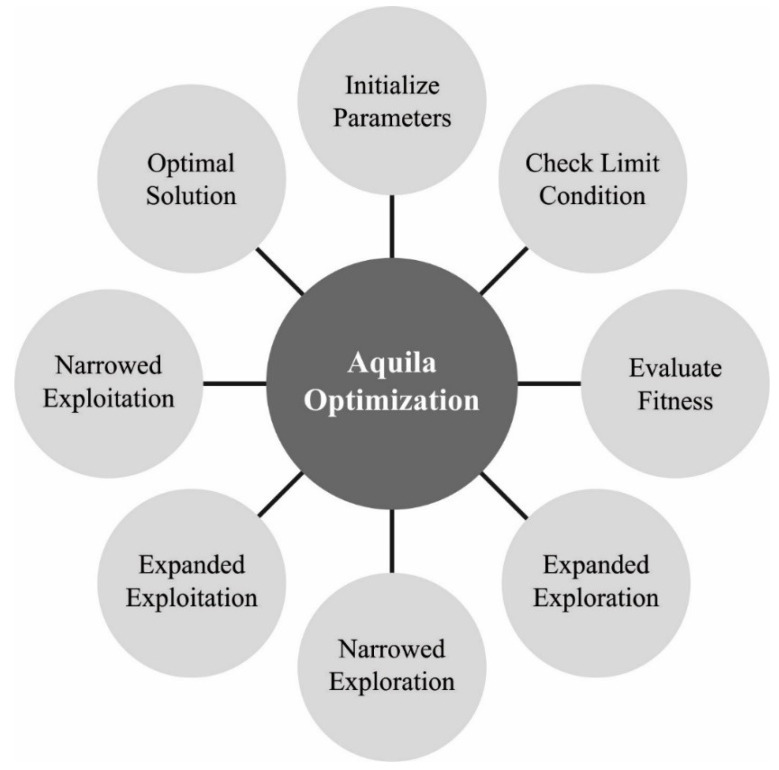
Flowchart of AO.

$UB_{j}$ and $LB_{j}$ indicate parameters of the searching space, $r_{1}\in \left[0,\;1\right]$ denotes a random number, and Dim shows the dimension of the agent. Next, exploration and exploitation are performed until the optimal solution is found. To perform exploration and exploitation, two strategies are employed. The initial strategy performs the exploration based on the average of agents $\left(X_{M}\right)$ and the finest agent $X_{b},$ as follows:

(7) $$X_{i}\left(t+1\right)=X_{b}\left(t\right)\times \left(1-\frac{t}{T}\right)+\left(X_{M}\left(t\right)-X_{b}\left(t\right)\times rand\right),\;$$

(8) $$X_{M}\left(t\right)=\frac{1}{N}\overset{N}{\underset{i=1}{\sum }}X\left(t\right),\forall j=1,2,\ldots ,Dim$$

where $\left(1-\frac{t}{T}\right)$ controls the search in the exploration stage, T indicates the maximal amount of iterations, and rand shows a random number within [0, 1]. Figure 2 showcases the flowchart of the AO method.

The next strategy used to perform the Levy flight (Leνy(D)) distribution and $X_{b}$ for updating the exploration capability of the solution is:

(9) $$X_{i}\left(t+1\right)=X_{b}\left(t\right)\times Levy\left(D\right)+X_{R}\left(t\right)+\left(y-x\right)\times rand$$

(10) $$Levy\;\left(D\right)=s\times \frac{u\times \sigma }{|v{|}^{\frac{1}{\beta }}},\sigma =\left(\frac{\Gamma \left(1+\beta \right)\times sine\left(\frac{\pi \beta }{2}\right)}{\Gamma \left(\frac{1+\beta }{2}\right)\times \beta \times 2\left(\frac{\beta -1}{2}\right)}\right)\;$$

where s=0.01 and β=1.5, u and v denote random numbers, and Γ indicates a constant number. In Equation (9), $X_{R}$ indicates an arbitrarily selected agent. Furthermore, y and x imitate the spiral shape, as given below:

(11) y=r× cos θ, x=r× sin θ 

(12) $$r=r_{2}+U\times D_{1},\;\theta =-\omega \times D_{1}+\theta_{1},\;\theta 1=\frac{3\times \pi }{2}\;$$

where ω=0.005, U=0.00565, $r_{2}\in \left[0,20\right]$ represents a random number, and $D_{l}$ indicates integer numbers from 1 to the length of the search domain.

An initial strategy is used to upgrade agents inside the exploitation stage based on $X_{b}$ and $X_{M}$, like exploration as follows:

(13) $$X_{i}\left(t+1\right)=\left(X_{b}\left(t\right)-X_{M}\left(t\right)\right)\times \alpha -rand+\left(\left(UB-LB\right)\times rand+LB\right)\times \delta$$

where α and δ signify the exploitation adjustment parameter and rand∈[0, 1] denotes a random number. In the next strategy of exploitation, the agent gets upgraded based on $X_{b}$, *Levy*, and the quality function QF:

(14) $$X_{i}\left(t+1\right)=QF\times X_{b}\left(t\right)-\left(G_{1}\times X\left(t\right)\times rand\right)-G_{2}\times Levy\left(D\right)+rand\times G_{1}$$

(15) $$QF\left(t\right)=t\frac{2\times rand\left(\right)-1}{{\left(1-T\right)}^{2}}$$

where *rand* () denotes a function that produces a random number and $G_{1}$ specifies distinct motions that are used to track the best individual solution, as follows:

(16) $$G_{1}=2\times rand\left(\right)-1,$$

where *rand* shows a random number. Given that, $G_{2}$ specifies reducing value from 2 to 0, and it is evaluated by Equation (17):

(17) $$G_{2}=2\times \left(1-\frac{t}{T}\right)$$

Algorithm 1 illustrates the fundamental steps of the AO.
**Algorithm 1:** Aquila optimizer (AO)Input: Describe the number of solutions N, the overall number of iterations T, and the dimension of every solution Dim. Set the initial value for the parameter of AO. Produce early population X. while (The ending criteria are not satisfied) do    $\mathrm{Calculate}\;\mathrm{the}\;\mathrm{fitness}\;\mathrm{value}\;\mathrm{for}\;\mathrm{every}\;X_{i}.$    $\mathrm{Find}\;\mathrm{the}\;\mathrm{optimal}\;\mathrm{individual}\;X_{b}\left(t\right)$    for (i=1,2…,N) do         If t≤$\left(\frac{2}{3}\right)*T$             $\mathrm{Upgrade}\;\mathrm{the}\;X_{i}$ based on Equation (7).             $\mathrm{If}\;\mathrm{the}\;\mathrm{FF}\;\left(Fit\right)\left(X_{1}\left(t+1\right)\right)<Fit\left(X\left(t\right)\right)$ then                              $X_{b}\left(t\right)=\left(X_{1}\left(t+1\right)\right)$                  $\mathrm{If}\;\mathrm{Fit}\;\left(X_{1}\left(t+1\right)\right)<Fit\left(X_{b}\left(t\right)\right)$ then                                 $X_{b}\left(t\right)=X_{1}\left(t+1\right)$                  End if             End if             $\mathrm{Upgrade}\;\mathrm{the}\;X_{i}$ based on Equation (9)             $\mathrm{If}\;\mathrm{Fit}\;\left(X_{2}\left(t+1\right)\right)<Fit\left(X\left(t\right)\right)$ then                              $X\left(t\right)=\left(X_{2}\left(t+1\right)\right)$                  $\mathrm{If}\;\mathrm{Fit}\;\left(X_{2}\left(t+1\right)\right)<Fit\left(X_{b}\left(t\right)\right)$ then                                 $X_{b}\left(t\right)=X_{2}\left(t+1\right)$                  End if             End if         Else             $\mathrm{Upgrade}\;\mathrm{the}\;X_{j}$ based on Equation (13).             $\mathrm{If}\;\mathrm{Fit}\;\left(X_{3}\left(t+1\right)\right)<Fit\left(X\left(t\right)\right)$ then                              $X\left(t\right)=\left(X_{3}\left(t+1\right)\right)$                  $\mathrm{if}\;\mathrm{Fit}\;\left(X_{3}\left(t+1\right)\right)<Fit\left(X_{b}\left(t\right)\right)$ then                                 $X_{b}\left(t\right)=X_{3}\left(t+1\right)$                  end if             end if             $\mathrm{Upgrade}\;X_{i}$ based on Equation (14).             $\mathrm{if}\;\mathrm{Fit}\;\left(X_{4}\left(t+1\right)\right)<Fit\left(X\left(t\right)\right)$ then                              $X\left(t\right)=\left(X_{4}\left(t+1\right)\right)$                  $\mathrm{if}\;\mathrm{Fit}\;\left(X_{4}\left(t+1\right)\right)<Fit\left(X_{b}\left(t\right)\right)$ then                                 $X_{b}\left(t\right)=X_{4}\left(t+1\right)$
                  end if             end if         End if    end for End while $\mathrm{Output}\;\mathrm{return}\;\left(X_{b}\right)$.

In the WMAO algorithm, the mutation was considered a significant technique to support the technique’s jump from local optimum [20]. In this phase, the wavelet mutating approach for enhancing the execution of the AO method is presented. Once the mutation probability P is set, every individual after the exploitation and exploration phase of the method will be getting a mutation chance via wavelet mutation strategy. Where rand<P, an individual executes Morlet wavelet mutation. The formula of mutation is:

(18) $$X_{i}^{new}\left(t\right)=\{\begin{array}{ll} X_{i}\left(t\right)+\sigma \left(UB-X_{i}\left(t\right)\right), & rand<0.5\\ X_{i}\left(t\right)+\sigma \left(X_{i}\left(t\right)-LB\right), & rand\geq 0.5 \end{array}\;$$

where $X_{i}\left(t\right)\left(i=1,2,\;\cdots ,\;N\right)$ denotes the *i*-th individual location in *t*-th generation, and *LB* and *UB* are the lower and upper bounds of the present search space. Correspondingly, σ represents the wavelet mutating coefficient. Its formula is [21]:

(19) $$\sigma =\frac{1}{\sqrt{\alpha }}\psi \left(\frac{\varphi }{\alpha }\right)$$

where $\psi \left(\varphi /\alpha \right)=e^{-{\left(\varphi /\alpha \right)}^{2}/2}\cdot \;\cos\;\left(5\varphi /\alpha \right)$ is the Morlet wavelet function, and 99% of its energy can be concentrated within −2.5 and 2.5, so *φ* signifies an arbitrary number within −2.5*α* and 2.5*α*. The a is the scaling parameter and its expression is [22]:

(20) $$\alpha =s\cdot {\left(\frac{1}{s}\right)}^{\left(1-\frac{t}{t\;\max\;}\right)}$$

where *s* indicates a given constant. The mutant individual $X_{i}^{new}$ is gained after the wavelet mutation function is completed, and greedy choice can be made among the original individual $X_{i}$ and mutant individual $X_{i}^{new}$, i.e.,

(21) $$X_{i}\left(t+1\right)=\{\begin{array}{ll} X_{i}^{new}\left(t\right), & f\left(X_{i}^{new}\left(t\right)\right)\leq f\left(X_{i}\left(t\right)\right)\\ X_{i}\left(t\right), & f\left(X_{i}^{new}\left(t\right)\right)>f\left(X_{i}\left(t\right)\right) \end{array}$$

This procedure assures that an individual with superior fitness will be entering the next iteration, thereby enhancing the convergence speed and optimization capability of the algorithm.

### 3.3. Process Involved in the WMAO-EAR Technique

In the presented WMAO-EAR technique, an FF is derived using distinct constraints, such as delay, energy, distance, and security. The main concept of the proposed algorithm is to decrease the communication distance between the selected nodes and CH [23]. It also focuses on decreasing the delay to transmit the information from one node to another. In contrast, network energy has to be higher, i.e., during data transmission, it needs to employ a small amount of energy. Finally, the node must tolerate the risk attained in the network. The objective function of the adapted CH demonstrated in Equation (22), while the value of η needs to depend on 0<η<1. Now, $v_{m}$ and $v_{n}$ indicate the operation, as demonstrated in Equations (23) and (24). The constraints on delay, energy, distance, and security are indicated as $\sigma_{1},$ $\sigma_{2},\;\;\sigma_{3,}$ and $\sigma_{4}$. The condition of this constraint is indicated as $\sigma_{1}+\sigma_{2}+\sigma_{3}+\sigma_{4}=1$. In Equation (24), $Y^{Z}-S_{s}$ denotes the distance between the normal and sink nodes.

(22) $$N_{n}=\eta v_{n}+\left(1-\eta \right)v_{m}$$

(23) $$v_{m}=\sigma_{1}*v_{i}^{dis}+\sigma_{2}*v_{i^{me}}+\sigma_{3}*v_{i^{de/}}+\sigma_{4}*v_{i^{\sec}}$$

(24) $$v_{n}=\frac{1}{b}\overset{b}{\underset{z=1}{\sum }}\|Y^{K}-S_{J}\|$$

Equation (25) illustrates the FF for distance, where $di_{J}\left(m\right)$ is related to packet transmission from the normal nodes to CH and CH to BS. Generally, $v_{i^{dis}}$ lies amongst [0, 1] and the value goes high when the distance between the normal node and CH is higher. Equations (26) and (27) demonstrate $v_{\left(m\right)}^{dis}$ and $v^{dis}\left(n\right)$, where $Y_{z}$ indicates the normal node in zth cluster, $G_{z}$ represents the CH of Zth cluster, the distance between the BS and CH is stated as $G_{Z}-S_{s},\;G_{Z}-Y_{y}$ symbolizes the distance between the normal node and CH, $Y_{z}-Y_{y}$ indicates the distance between two normal nodes, and $M_{z}$ and $M_{y}\vee$ represent the node amount that eliminates the Zth and yth clusters.

(25) $$v_{i}^{dis}=\frac{v_{\left(m\right)}^{dis}}{v_{\left(n\right)}^{dis}}\;$$

(26) $$v_{\left(m\right)}^{dis}=\overset{M_{z}}{\underset{z=1}{\sum }}\left[\left\|G_{x}-S_{s}\|+\overset{M_{y}}{\underset{y=1}{\sum }}\|G_{Z}-Y_{y}\right\|\right]$$

(27) $$v_{\left(n\right)}^{dis}=\overset{M_{z}}{\underset{z=1}{\sum }}\overset{M_{y}}{\underset{y=1}{\sum }}\|Y_{z}-Y_{y}\|$$

Equation (28) illustrates the FF of energy. The value $v_{i^{ene}}\;$is greater than 1 and the whole CH cumulative $v_{\left(m\right)}^{ene}$ and $v_{\left(n\right)}^{ene}$ concern the maximum energy value and the greater amount of CH.

(28) $$v_{i}^{ene}=\frac{v_{\left(m\right)}^{ene}}{v_{\left(n\right)}^{ene}}\;$$

Equation (29) shows the FF of delay $v_{i}^{del}$ that ranges within [0, 1]. $v_{i}^{del}$ is directly proportional to each node inherent in the cluster. Consequently, the delay gets minimalized when the CH has a decreased number of nodes. The numerator indicates the high amount of CH and the denominator $M_{M}$ indicates the total amount of nodes in the WSN.

(29) $$v_{i}^{del}=\frac{\;\max\;{\left(\|G_{z}-Y_{z}\|\right)}_{z=1}^{M_{c}}}{M_{M}}$$

## 4. Results and Discussion

**Figure 3:**
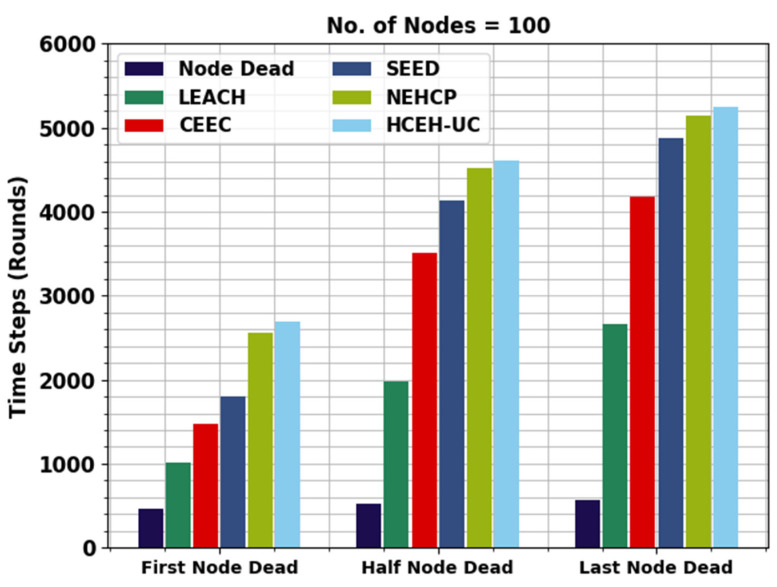
Lifetime analysis of WMAO-EAR approach under NC of 100.

This section inspects the routing performance of the WMAO-EAR method on WSNs. The presented WMAO-EAR model is tested under two node counts (NC) of 100 and 300. Table 1 provides a lifetime inspection of the WMAO-EAR model with existing methods in terms of FND, HND, and LND [24]. Figure 3 exhibits a brief lifetime assessment of the WMAO-EAR method under NC of 100. The results implied that the WMAO-EAR model attained an extended lifetime. For example, concerning FND, the WMAO-EAR model offered an increased FND of 1500 rounds, whereas the LEACH, CE-EC, SEED, NEH-CP, and HCEHUC models reached reduced FND of 466, 1000, 990, 780, and 2563 rounds, respectively. In terms of HND, the WMAO-EAR technique presented an increased HND of 4750 rounds, whereas the LEACH, CE-EC, SEED, NEH-CP, and HCEHUC techniques reached a reduced HND of 531, 4328, 3269, 2251, and 4525 rounds, respectively. Finally, in terms of LND, the WMAO-EAR algorithm provided an increased LND of 5432 rounds, while the LEACH, CE-EC, SEED, NEH-CP, and HCEHUC techniques achieved reduced LND of 275, 5230, 5072, 4500, and 5140 rounds, respectively.

**Figure 4:**
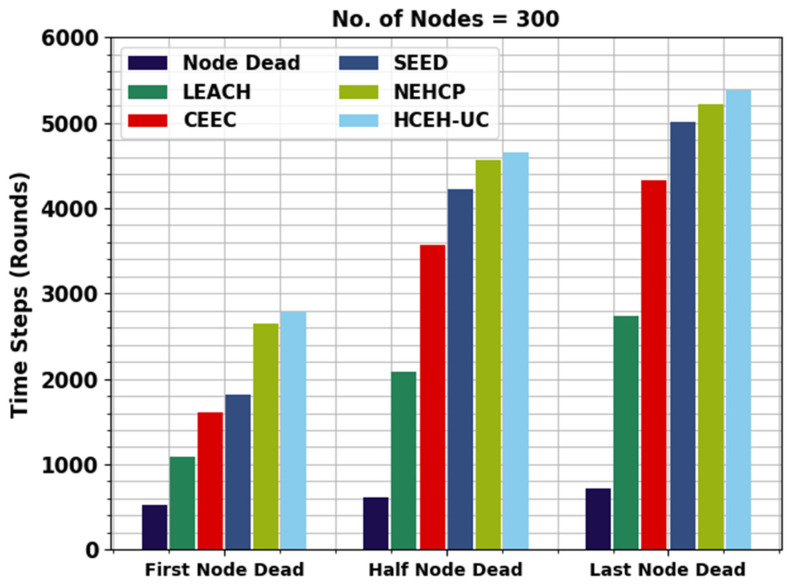
Lifetime analysis of WMAO-EAR approach under NC of 300.

Figure 4 displays the detailed lifetime assessment of the WMAO-EAR approach under NC of 300. The results denote the WMAO-EAR method obtained an extended lifetime. For example, in terms of FND, the WMAO-EAR approach has rendered an increased FND of 2786 rounds, whereas the LEACH, CE-EC, SEED, NEH-CP, and HCEHUC approaches attained reduced FND of 528, 1091, 1610, 1814, and 2651 rounds, respectively. For HND, the WMAO-EAR method and HCEHUC models reached reduced HND of 616, 2088, 3566, 4221, and 4562 rounds, respectively. Finally, in terms of LND, the WMAO-EAR approach gave an increased LND of 5387 rounds, where the LEACH, CE-EC, SEED, NEH-CP, and HCEHUC methods reached reduced LND of 715, 2736, 5072, 4327, and 5017 rounds, respectively.

**Figure 5:**
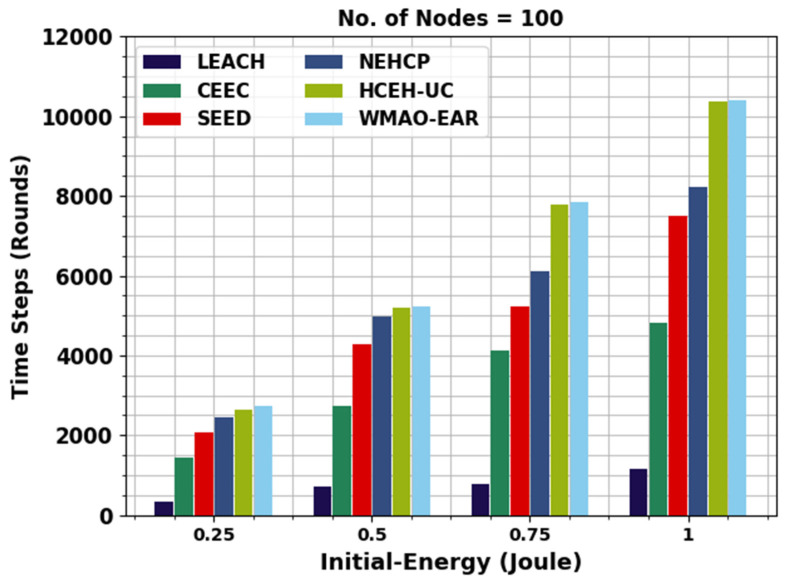
EU analysis of WMAO-EAR approach under NC of 100.

Table 2 presents a detailed energy utilization (EU) examination of the WMAO-EAR method with other approaches. Figure 5 provides the initial EU inspection of the WMAO-EAR model with compared methods on NC of 100. The figure shows that the WMAO-EAR model achieved EU in the latter rounds of execution. For example, with 0.25 J energy, the WMAO-EAR method ran to 2722 rounds, whereas the LEACH, CE-EC, SEED, NEH-CP, and HCEHUC models executed for 340, 1436, 2067, 2445, and 2649 rounds, respectively. Concurrently, with 0.5 J energy, the WMAO-EAR approach ran to 5213 rounds, whereas the LEACH, CE-EC, SEED, NEH-CP, and HCEHUC approaches executed for 702, 2748, 4287, 4985, and 5211 rounds, respectively.

**Figure 6:**
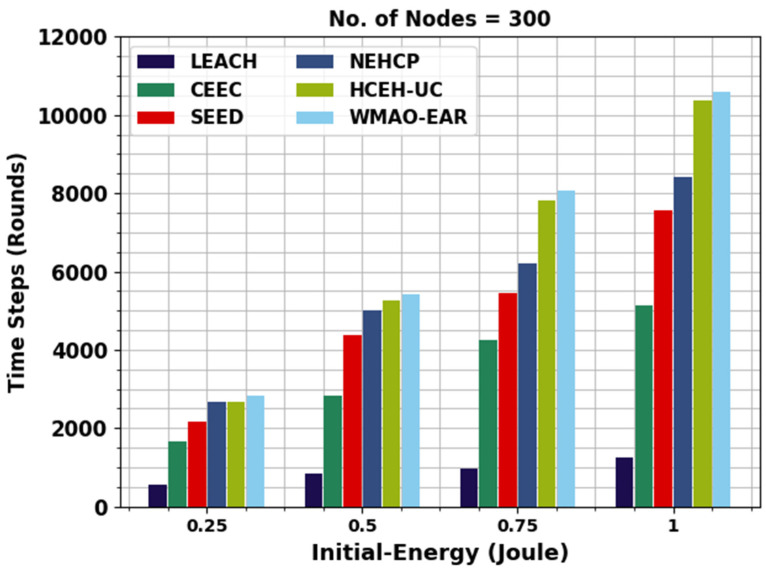
EU analysis of WMAO-EAR approach under NC of 300.

Figure 6 offers the initial EU review of the WMAO-EAR technique with compared methods on NC of 300. The figure shoes that the WMAO-EAR approach obtained EU in the latter rounds of execution. For example, with 0.25 J energy, the WMAO-EAR algorithm runs to 2837 rounds, whereas the LEACH, CE-EC, SEED, NEH-CP, and HCEHUC approaches execute 558, 1656, 2161, 2676, and 2661 rounds, respectively. Concurrently, with 0.5 J energy, the WMAO-EAR methodology runs to 5423 rounds, whereas the LEACH, CE-EC, SEED, NEH-CP, and HCEHUC techniques execute 846, 2815, 4390, 4997, and 5259 rounds, respectively.

**Figure 7:**
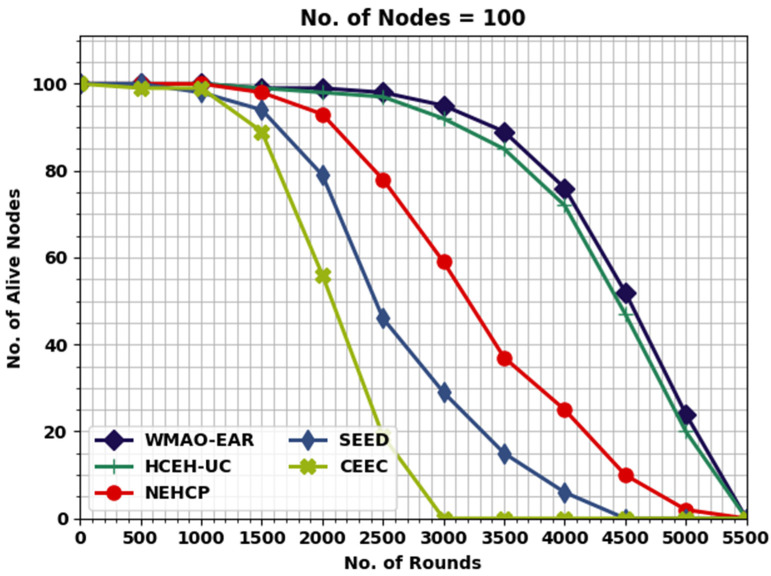
NOAN analysis of WMAO-EAR approach under NC of 100.

Table 3 provides a detailed NOAN evaluation of the WMAO-EAR model with other existing models. Figure 7 highlights the comparative NOAN inspection of the WMAO-EAR model. With 2000 rounds, the NOAN offered by the WMAO-EAR model is 99, whereas the CE-EC, SEED, NEH-CP, and HCEHUC models obtained reduced NOAN of 98, 93, 79, and 56, respectively. Likewise, with 2500 rounds, the NOAN presented by the WMAO-EAR approach is 98, whereas the CE-EC, SEED, NEH-CP, and HCEHUC techniques attained reduced NOAN of 97, 78, 46, and 19, respectively. With 3000 rounds, the NOAN presented by the WMAO-EAR algorithm is 95, whereas the CE-EC, SEED, NEH-CP, and HCEHUC methodologies attained reduced NOAN of 92, 59, 29, and 0, respectively.

**Figure 8:**
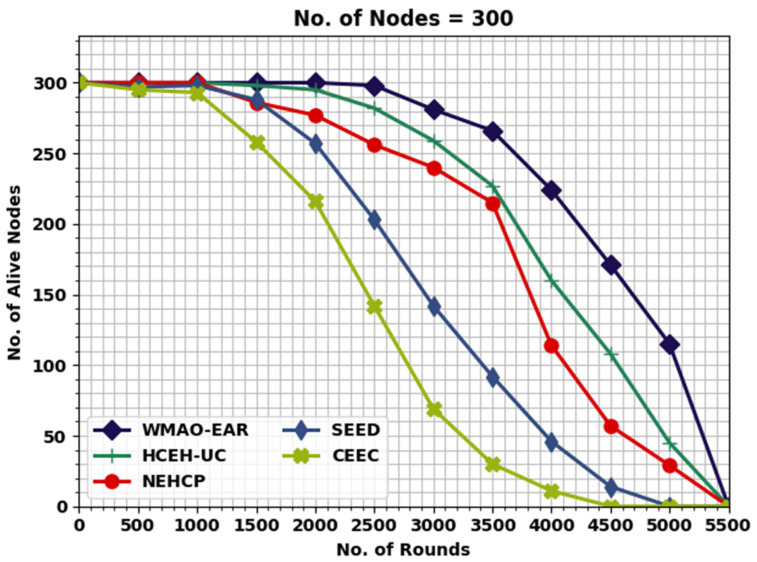
NOAN analysis of WMAO-EAR approach under NC of 300.

Figure 8 illustrates the comparative NOAN study of the WMAO-EAR approach. With 2000 rounds, the NOAN 300 rendered by the WMAO-EAR technique is 300, whereas the CE-EC, SEED, NEH-CP, and HCEHUC methods attained reduced NOAN of 295, 277, 257, and 216, respectively. With 2500 rounds, the NOAN granted by the WMAO-EAR model is 298, whereas the CE-EC, SEED, NEH-CP, and HCEHUC approaches achieved reduced NOAN of 282, 256, 203, and 142, respectively. With 3000 rounds, the NOAN offered by the WMAO-EAR method is 281, whereas the CE-EC, SEED, NEH-CP, and HCEHUC models attained reduced NOAN of 259, 240, 142, and 69, respectively.

**Figure 9:**
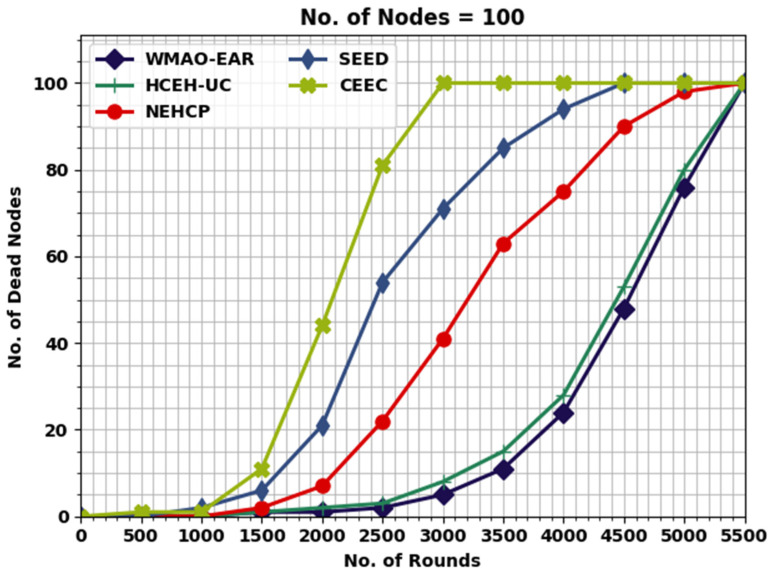
NODN analysis of WMAO-EAR approach under NC of 100.

A comparative NODN assessment of the WMAO-EAR model with other models is provided in Table 4. Figure 9 illustrates a detailed NODN investigation of the WMAO-EAR model with other approaches. The outcomes indicated that the WMAO-EAR method reached maximum outcome with minimal NODN values. For example, with 2000 rounds, the WMAO-EAR model gained a lower NODN of 1, whereas the CE-EC, SEED, NEH-CP, and HCEHUC models attained higher NODN of 2, 7, 21, and 44, respectively. Finally, with 3000 rounds, the WMAO-EAR method gained a lower NODN of 5, whereas the CE-EC, SEED, NEH-CP, and HCEHUC approaches attained higher NODN of 8, 41, 71, and 100, respectively.

**Figure 10:**
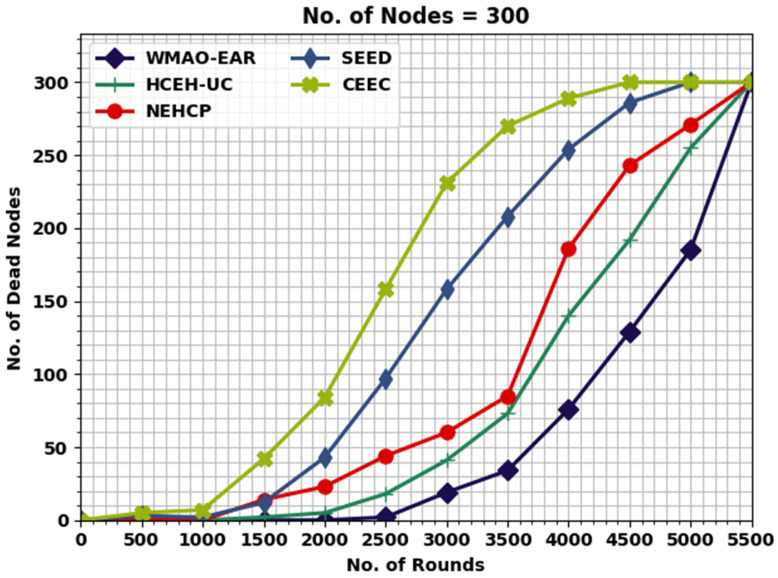
NODN analysis of WMAO-EAR approach under NC of 300.

Figure 10 illustrates a comprehensive NODN investigation of the WMAO-EAR technique with other models. The outcomes show that the WMAO-EAR approach attained maximum outcome with minimal NODN values. For instance, with 2000 rounds, the WMAO-EAR approach gained a lower NODN of 0, whereas the CE-EC, SEED, NEH-CP, and HCEHUC approaches attained higher NODN of 5, 23, 43, and 84, respectively.

Eventually, with 2500 rounds, the WMAO-EAR method attained a lower NODN of 2, whereas the CE-EC, SEED, NEH-CP, and HCEHUC approaches attained higher NODN of 18, 44, 97, and 158, respectively. These experimental values show the enhancements of the WMAO-EAR technique over other existing models.

## 5. Conclusions

In this study, a new WMAO-EAR approach was devised for effective and energy-aware wireless communication. The presented WMAO-EAR technique aims to accomplish an energy-aware routing process in WSNs. To do this, the WMAO-EAR technique initially derives the WMAO algorithm for the integration of wavelet mutation with the AO algorithm. An FF is derived using distinct constraints, such as delay, energy, distance, and security. Once the mutation probability P is set, every individual next to the exploitation and exploration stage process has a probability of mutation using the wavelet mutation process. To demonstrate the enhanced performance of the WMAO-EAR technique, a comprehensive simulation analysis has been presented. The experimental results establish the superiority of the WMAO-EAR method over other recent approaches. In the future, lightweight cryptographic solutions can be applied to boost secure communication.

## Figures and Tables

**Table 1:** Lifetime analysis of WMAO-EAR approach with existing algorithms under NC of 100 and 300.

Time Steps (Rounds)						
Dead Nodes	LEACH	CE-EC	SEED	NEH-CP	HCEHUC	WMAO-EAR
**Nodes = 100**						
First Node Dead	466	1000	990	780	2563	1500
Half Node Dead	531	4328	3269	2251	4525	4750
Last Node Dead	575	5230	5072	4500	5140	5432
**Nodes = 300**						
First Node Dead	528	1091	1610	1814	2651	2786
Half Node Dead	616	2088	3566	4221	4562	4647
Last Node Dead	715	2736	4327	5017	5217	5387

**Table 2:** EU analysis of WMAO-EAR approach with existing algorithms under NC of 100 and 300.

Time Steps (Rounds)						
Initial Energy (J)	LEACH	CE-EC	SEED	NEH-CP	HCEHUC	WMAO-EAR
**Nodes = 100**						
0.25	340	1436	2067	2445	2649	2722
0.5	702	2748	4287	4985	5211	5213
0.75	765	4136	5240	6101	7788	7862
1	1166	4825	7499	8211	10362	10411
**Nodes = 300**						
0.25	558	1656	2161	2676	2661	2837
0.5	846	2815	4390	4997	5259	5423
0.75	966	4261	5439	6192	7804	8082
1	1249	5138	7570	8415	10376	10585

**Table 3:** NOAN analysis of WMAO-EAR approach with existing algorithms under NC of 100 and 300.

Alive Nodes					
Rounds	WMAO-EAR	CE-EC	SEED	NEH-CP	HCEHUC
**Nodes = 100**					
0	100	100	100	100	100
500	100	100	100	100	99
1000	100	100	100	98	99
1500	99	99	98	94	89
2000	99	98	93	79	56
2500	98	97	78	46	19
3000	95	92	59	29	0
3500	89	85	37	15	0
4000	76	72	25	6	0
4500	52	47	10	0	0
5000	24	20	2	0	0
5500	0	0	0	0	0
**Nodes = 300**					
0	300	300	300	300	300
500	300	300	300	297	295
1000	300	300	300	298	293
1500	300	298	286	288	258
2000	300	295	277	257	216
2500	298	282	256	203	142
3000	281	259	240	142	69
3500	266	227	215	92	30
4000	224	160	114	46	11
4500	171	108	57	14	0
5000	115	45	29	0	0
5500	0	0	0	0	0

**Table 4:** NODN analysis of WMAO-EAR approach with existing algorithms under NC of 100 and 300.

Dead Nodes					
Rounds	WMAO-EAR	HCEHUC	NEH-CP	SEED	CE-EC
**Nodes = 100**					
0	0	0	0	0	0
500	0	0	0	0	1
1000	0	0	0	2	1
1500	1	1	2	6	11
2000	1	2	7	21	44
2500	2	3	22	54	81
3000	5	8	41	71	100
3500	11	15	63	85	100
4000	24	28	75	94	100
4500	48	53	90	100	100
5000	76	80	98	100	100
5500	100	100	100	100	100
**Nodes = 300**					
0	0	0	0	0	0
500	0	0	0	3	5
1000	0	0	0	2	7
1500	0	2	14	12	42
2000	0	5	23	43	84
2500	2	18	44	97	158
3000	19	41	60	158	231
3500	34	73	85	208	270
4000	76	140	186	254	289
4500	129	192	243	286	300
5000	185	255	271	300	300
5500	300	300	300	300	300

## Data Availability

Data sharing does not apply to this article as no datasets were generated.
